# Predicting Antibiotic Resistance in Gram-Negative Bacilli from Resistance Genes

**DOI:** 10.1128/AAC.02462-18

**Published:** 2019-03-27

**Authors:** G. Terrance Walker, Julia Quan, Stephen G. Higgins, Nikhil Toraskar, Weizhong Chang, Alexander Saeed, Vadim Sapiro, Kelsey Pitzer, Natalie Whitfield, Bert K. Lopansri, Mary Motyl, Daniel Sahm

**Affiliations:** a OpGen, Inc., Gaithersburg, Maryland, USA; b Intermountain Medical Center, Murray, Utah, USA; c University of Utah, Salt Lake City, Utah, USA; d Merck & Co., Inc., Whitehouse Station, New Jersey, USA; e IHMA, Inc., Schaumburg, Illinois, USA

**Keywords:** PCR, antibiotic resistance, resistance genes

## Abstract

We developed a rapid high-throughput PCR test and evaluated highly antibiotic-resistant clinical isolates of Escherichia coli (*n* = 2,919), Klebsiella pneumoniae (*n* = 1,974), Proteus mirabilis (*n* = 1,150), and Pseudomonas aeruginosa (*n* = 1,484) for several antibiotic resistance genes for comparison with phenotypic resistance across penicillins, cephalosporins, carbapenems, aminoglycosides, trimethoprim-sulfamethoxazole, fluoroquinolones, and macrolides. The isolates originated from hospitals in North America (34%), Europe (23%), Asia (13%), South America (12%), Africa (7%), or Oceania (1%) or were of unknown origin (9%). We developed statistical methods to predict phenotypic resistance from resistance genes for 49 antibiotic-organism combinations, including gentamicin, tobramycin, ciprofloxacin, levofloxacin, trimethoprim-sulfamethoxazole, ertapenem, imipenem, cefazolin, cefepime, cefotaxime, ceftazidime, ceftriaxone, ampicillin, and aztreonam. Average positive predictive values for genotypic prediction of phenotypic resistance were 91% for E. coli, 93% for K. pneumoniae, 87% for P. mirabilis, and 92% for P. aeruginosa across the various antibiotics for this highly resistant cohort of bacterial isolates.

## Abstract (precis)

We developed a rapid high-throughput PCR test and evaluated highly antibiotic-resistant clinical isolates of Escherichia coli (*n* = 2,919), Klebsiella pneumoniae (*n* = 1,974), Proteus mirabilis (*n* = 1,150), and Pseudomonas aeruginosa (*n* = 1,484) for several antibiotic resistance genes for comparison with phenotypic resistance across penicillins, cephalosporins, carbapenems, aminoglycosides, trimethoprim-sulfamethoxazole, fluoroquinolones, and macrolides. The isolates originated from hospitals in North America (34%), Europe (23%), Asia (13%), South America (12%), Africa (7%), or Oceania (1%) or were of unknown origin (9%).

## INTRODUCTION

Two million Americans are infected with antibiotic-resistant bacteria each year, resulting in 23,000 deaths (1). Associated annual direct health care costs exceed $20 billion. The health care crisis is fueled by Gram-negative multidrug-resistant organisms, including carbapenem-resistant *Enterobacteriaceae* (CRE), which have been reported in 47 of the 50 United States and with outbreaks being reported in major cities (2).

Health care providers lack timely guidance for effective treatment of antibiotic-resistant infections. Phenotypic antimicrobial susceptibility testing (AST) is the standard method to guide antibiotic use in clinical practice for culture-positive bacterial infections. Conventional growth-based organism identification and AST are comprehensive and cost-effective but slow, with a typical total turnaround time of 48 h, while emerging growth-based methods offer shorter turnaround times (3, 4). As an alternative to growth-based methods, molecular diagnostic tests rapidly detect antibiotic resistance genes from cultured bacterial isolates or primary specimens within hours of collection from the patient. However, molecular tests often lack enough antibiotic resistance genes for comprehensive prediction of phenotypic antibiotic resistance for multiple antibiotic classes.

This study evaluated more than 7,500 isolates of Escherichia coli, Klebsiella pneumoniae, Proteus mirabilis, and Pseudomonas aeruginosa to predict phenotypic resistance to several antibiotic classes through detection of resistance genes using a high-throughput multiplex PCR test and statistical modeling. The bacterial isolates originated from hospitals spanning six continents.

## RESULTS

### Clinical isolates and phenotypic AST.

Clinical isolates of antibiotic-resistant E. coli (*n* = 2,919), K. pneumoniae (*n* = 1,974), P. mirabilis (*n* = 1,150), and P. aeruginosa (*n* = 1,484) were collected by the central clinical microbiology laboratory at the Intermountain Medical Center (Murray, UT) and through the Merck Study for Monitoring Antimicrobial Resistance Trends (SMART), which has tracked trends in the antibiotic susceptibility of 250,000 bacterial isolates collected from 192 hospitals in 54 countries across the world since 2002. The SMART isolates were chosen from the Merck biorepository based on reported phenotypic resistance to several antibiotics and broad geographic origin (Table 1).

**TABLE 1:** Geographic origin of isolates

Origin	No. of isolates				Distribution (%)
	E. coli	K. pneumoniae	P. mirabilis	P. aeruginosa	
North America	1,576	472	57	450	34
Europe	394	515	461	330	23
Asia	204	300	275	224	13
South America	167	367	153	251	12
Africa	111	189	144	100	7
Oceania	30	13	17	44	1
Unknown	437	118	43	85	9
					
Total	2,919	1,974	1,150	1,484	

The isolates underwent PCR genotyping and phenotypic AST. We detected high levels of phenotypic resistance to several antibiotics, with most isolates exhibiting resistance to multiple antibiotic classes (Table 2). High levels of phenotypic resistance allowed us to associate resistance genes with phenotypic resistance to several antibiotics for the four organisms using a relatively small number of isolates. AST assignments of susceptible are reported as such, while resistant and intermediate assignments were considered nonsusceptible and are reported as resistant in this study, with slight exceptions for cefepime and cefotaxime. AST assignments of susceptible and susceptible dose dependent (SDD) were grouped together as susceptible for cefepime. The cefotaxime MIC levels on the MicroScan AST panel allowed interpretation of only susceptible/intermediate versus resistant, based on cefotaxime breakpoints from the Clinical and Laboratory Standards Institute (5).

**TABLE 2:** Percentage of isolates with nonsusceptible phenotypes per antibiotic

Antibiotic	% of isolates			
	E. coli (*n* = 2,919)	K. pneumoniae (*n* = 1,974)	P. mirabilis (*n* = 1,150)	P. aeruginosa (*n* = 1,484)
Gentamicin	33	58	40	60
Tobramycin	46	76	38	51
Ciprofloxacin	68	81	58	67
Levofloxacin	67	72	47	69
Trimethoprim-sulfamethoxazole	58	75	63	Intrinsic resistance
Imipenem	7	39	Intrinsic resistance	64
Ertapenem	13	51	1	Intrinsic resistance
Cefazolin	74	90	49	Intrinsic resistance
Cefepime	68	84	29	69
Cefotaxime	71	86	41	Intrinsic resistance
Ceftazidime	55	84	22	62
Ceftriaxone	71	87	41	Intrinsic resistance
Ampicillin	88	Intrinsic resistance	72	Intrinsic resistance
Aztreonam	67	84	13	64

### Predicting antibiotic resistance from resistance genes.

We developed a high-throughput multiplex PCR test for several resistance genes encoding penicillinases, cephalosporinases, carbapenemases, AmpC beta-lactamases, aminoglycoside-modifying enzymes, ribosomal methyltransferases, dihydrofolate reductase, plasmid-mediated quinolone resistance, macrolide-modifying enzymes, sulfonamide resistance, plasmid-mediated efflux pumps, and tetracycline and macrolide efflux (Table 3). OpGen offers the high-throughput multiplex PCR test as a commercial testing service for analysis of bacterial isolates. The resistance genes were chosen based on a review of the scientific literature and surveys of resistance gene databases (6, 7). PCR assays specific for gene variants at the amino acid positions indicated in Table 3 (e.g., for SHV, TEM, *gyrA*, *parC*) or homologous sequence regions shared by different gene subtypes within a family of closely related resistance genes were designed.

**TABLE 3:** Antibiotic resistance genes tested by PCR

Antibiotic class and resistance gene
Aminoglycosides
*aac(3)*
*aac(6')*
*aadA3*
*aadA5*
*aadA6*
*aadA7*
*aadA9*
*aadA12*
*aadA16*
*ant(2”)*
*ant(3′)*
*aph(3′)*
*aph(4)*
*aph(6)*
*strA*
*strB*
*armA*
*rmtB*
*rmtF*
Beta-lactams
*bla*_ACC-1_
*bla*_ACT-1_
*bla*_BEL-1_
*bla*_BES-1_
*bla*_CMY-2_
*bla*_CTX-M-1_
*bla*_CTX-M-2_
*bla*_CTX-M-8_
*bla*_CTX-M-9_
*bla*_DHA-1_
*bla*_FOX-1_
*bla*_GES-1_
*bla*_GIM-1_
*bla*_IMI-1_
*bla*_IMP-1_
*bla*_KPC-2_
*bla*_MIR-1_
*bla*_MOX-1_
*bla*_NDM-1_
*bla*_NMC-A_
*bla*_OXA-1_
*bla*_OXA-2_
*bla*_OXA-9_
*bla*_OXA-10_
*bla*_OXA-18_
*bla*_OXA-23_
*bla*_OXA-24_
*bla*_OXA-45_
*bla*_OXA-48_
*bla*_OXA-50_
*bla*_OXA-51_
*bla*_OXA-54_
*bla*_OXA-55_
*bla*_OXA-58_
*bla*_PER-1_
*bla*_PSE-1_
*bla*_SFC-1_
*bla*_SHV_^*a*^
*bla*_SIM-1_
*bla*_SME-1_
*bla*_SPM-1_
*bla*_TEM_^*a*^
*bla*_TLA-1_
*bla*_VEB-1_
*bla*_VIM-1_
Colistin, *mcr-1*
Fluoroquinolones
E. coli *gyrA*-83^*b*^
E. coli *gyrA*-87^*b*^
E. coli *parC*-240^*b*^
K. pneumoniae *gyrA*-83^*b*^
K. pneumoniae *gyrA*-87^*b*^
K. pneumoniae *parC*-240^*b*^
P. aeruginosa *gyrA*-83^*b*^
P. aeruginosa *gyrA*-87^*b*^
P. aeruginosa *parC*-87^*b*^
*oqxA*
*oqxB*
*qnrA1*
*qnrA3*
*qnrB1*
*qnrB2*
*qnrB10*
*qnrB11*
*qnrB13*
*qnrB21*
*qnrB22*
*qnrB27*
*qnrB31*
*qnrD1*
*qnrS1*
*qnrS2*
Macrolides
*ere*(A)
*ere*(B)
*erm*(B)
*mph*(A)
*mph*(D)
*mph*(E)
*msr*(E)
*tet*(A)
*tetA*(B)
*tetA*(G)
*tetAJ*
Phenicol, *floR*
Sulfonamides
*sul1*
*sul2*
*sul3*
Trimethoprim
*dfrA1*
*dfrA5*
*dfrA7*
*dfrA8*
*dfrA12*
*dfrA14*
*dfrA15*
*dfrA16*
*dfrA17*
*dfrA18* and *dfr19*
*dfrA23*
*dfrA27*
*dfrB1* and *dfr2a*
*dfrB2*
*dhfrB5*
Vancomycin, *vanA*

a The PCR assays for SHV detect several sequence variants at amino acid positions 156, 238, and 240. The PCR assays for TEM detect several sequence variants at amino acid positions 104, 164, 238, and 240. These SHV and TEM variants are associated with wild-type penicillin resistance and ESBL phenotypes.

b The PCR assays for *gyrA* and *parC* detect wild-type and mutant variants at the indicated amino acid positions for E. coli, K. pneumoniae, and P. aeruginosa.

The high-throughput multiplex PCR test uses automated DNA extraction and a microfluidic PCR system capable of analyzing 96 samples with 96 separate two-plex PCR assays per test run (18,432 PCR results per test run). DNA extraction and PCR require a total hands-on time of approximately 2 h, with the total turnaround time being approximately 5 h for 96 isolates.

We used the high-throughput multiplex PCR test to evaluate the isolates for resistance genes and generated statistical generalized linear models to identify resistance genes associated with phenotypic antibiotic resistance for each antibiotic-organism combination. Agreement between resistance genes and phenotypic resistance was evaluated through a series of stepwise gene additions/eliminations and 10-fold cross validation, repeated 30 times. Predictive resistance genes were chosen based on the highest cross-validation agreement with phenotypic AST and the smallest agreement variance. When generalized linear models lacked statistical significance, phenotypic resistance was predicted using simpler binary models based on just the presence of resistance genes.

Table 4 summarizes the performance of the generalized linear models, using the resistance genes for prediction of phenotypic resistance, in terms of the sensitivity, specificity, positive predictive value (PPV), negative predictive value (NPV), and the agreement of the genotypic prediction with the phenotypic AST result. Agreement represents the correct prediction of phenotypic resistance or the correct inference of phenotypic susceptibility in the absence of predicted resistance. Most generalized linear models predicted phenotypic resistance with a high sensitivity, which allowed the accurate inference of phenotypic susceptibility in the absence of predicted resistance. The simpler binary models (Table 5) accurately predicted phenotypic resistance in the presence of resistance genes but did not enable the accurate inference of phenotypic susceptibility in the absence of predicted resistance. In other words, the generalized linear models were more sensitive than the binary models for prediction of phenotypic resistance, although the binary models accurately predicted phenotypic resistance in the presence of resistance genes. For example, the binary model predicted phenotypic imipenem resistance across the 1,484 P. aeruginosa isolates with a sensitivity of only 32%, although the PPV was 94% for the subset of P. aeruginosa isolates harboring the carbapenem resistance genes used by the binary model (Table 5). Moreover, as related specifically to the carbapenem resistance gene VIM, 193 of the 1,484 P. aeruginosa isolates harbored a VIM gene, with 190 of the 193 VIM-positive isolates exhibiting phenotypic resistance to imipenem for a PPV of 98% but a sensitivity of only 20%, since 946 of the 1,484 P. aeruginosa isolates exhibited phenotypic resistance to imipenem.

**TABLE 4:** Performance of genotypic generalized linear models for prediction of phenotypic antibiotic resistance in E. coli, K. pneumoniae, P. mirabilis, and P. aeruginosa

Bacterium and antibiotic	Sensitivity (%)	Specificity (%)	% agreement (95% CI^*a*^)	PPV (%)	NPV (%)
E. coli (*n* = 2,919)					
Aztreonam	94	83	90 (89–91)	92	86
Cefazolin	90	91	91 (90–92)	97	77
Cefepime	88	91	89 (88–90)	95	79
Cefotaxime	93	92	92 (91–93)	96	84
Ceftazidime	80	83	81 (80–83)	86	77
Ceftriaxone	94	91	93 (92–94)	96	86
Ciprofloxacin	95	91	94 (93–95)	96	90
Levofloxacin	96	90	94 (93–95)	95	93
Gentamicin	87	95	92 (91–93)	90	93
Tobramycin	91	93	92 (91–93)	91	92
Trimethoprim-sulfamethoxazole	90	88	89 (88–90)	92	86
Piperacillin	95	88	94 (93–95)	98	72
K. pneumoniae (*n* = 1,974)					
Aztreonam	87	85	86 (85–88)	97	54
Cefazolin	100	0	90 (88–91)	90	NA^*b*^
Cefepime	88	85	88 (86–89)	97	57
Cefotaxime	90	86	89 (88–90)	98	56
Ceftazidime	88	77	86 (85–88)	95	55
Ceftriaxone	89	85	89 (87–90)	98	55
Ciprofloxacin	93	80	91 (89–92)	95	73
Levofloxacin	89	81	87 (95–88)	92	74
Imipenem	85	88	87 (85–88)	82	90
Gentamicin	84	89	86 (84–87)	91	80
Tobramycin	90	86	89 (88–91)	95	74
Trimethoprim-sulfamethoxazole	94	72	89 (87–90)	91	80
P. mirabilis (*n* = 1,150)					
Ampicillin	83	94	86 (84–88)	97	68
Cefazolin	83	63	73 (70–75)	68	79
Cefotaxime	83	94	90 (88–91)	90	89
Ceftazidime	73	94	89 (88–91)	78	93
Ceftriaxone	83	94	89 (87–90)	91	88
Gentamicin	78	95	88 (86–90)	91	86
Tobramycin	69	96	85 (83–87)	91	83
Trimethoprim-sulfamethoxazole	87	82	85 (83–87)	89	79
Piperacillin	89	88	88 (86–90)	92	84
P. aeruginosa (*n* = 1,484)					
Ciprofloxacin	86	82	84 (82–86)	91	74
Levofloxacin	83	81	82 (80–84)	91	69

a CI, confidence interval.

b NA, not available.

**TABLE 5:** Performance of binary models

Species	Antibiotic	No. of isolates:			PPV (%)
		Total	Predicted to be resistant	Predicted to be resistant and found to be phenotypically resistant	
E. coli	Ampicillin	2,919	2,429	2,393	99
E. coli	Imipenem	2,919	144	111	77
E. coli	Meropenem	2,919	144	104	72
E. coli	Ertapenem	2,919	211	160	76
K. pneumoniae	Ertapenem	1,974	795	734	92
K. pneumoniae	Meropenem	1,974	800	669	84
P. mirabilis	Aztreonam	1,150	56	46	82
P. mirabilis	Cefepime	1,150	257	229	89
P. aeruginosa	Aztreonam	1,484	164	139	85
P. aeruginosa	Ceftazidime	1,484	337	317	94
P. aeruginosa	Gentamicin	1,484	542	500	92
P. aeruginosa	Tobramycin	1,484	542	493	91
P. aeruginosa	Imipenem	1,484	322	303	94
P. aeruginosa	Meropenem	1,484	317	299	94

The isolates exhibited a balanced distribution of phenotypes for several antibiotics (Table 2), which enabled statistically significant prediction of phenotypic resistance from PCR results (e.g., for E. coli and gentamicin), as shown in Table 4. The modeled PCR results also accurately predicted phenotypic resistance for antibiotic-organism combinations with a less balanced distribution of phenotypes (e.g., E. coli and ampicillin), although with more limited statistical significance. False-positive and false-negative predictions of phenotypic resistance were comparable without appreciable bias within each organism-antibiotic combination.

## DISCUSSION

The resistance genes in Table 3 effectively covered the resistance mechanisms for the isolates in this study and could be used for the sensitive prediction of phenotypic resistance with generalized linear models, which allowed the inference of phenotypic susceptibility in the absence of predicted resistance (e.g., for E. coli and gentamicin). The binary models effectively predicted phenotypic resistance based on the presence of resistance genes but did not have sufficient sensitivity to infer phenotypic susceptibility in the absence of resistance genes (e.g., for P. aeruginosa and imipenem) because the binary models lacked full coverage of resistance mechanisms. For example, carbapenem resistance often derives from beta-lactamase resistance genes, which are readily detected by PCR, as in this study. However, carbapenem resistance also derives from a wide array of regulatory changes for AmpC enzymes, pumps, and porins, especially in P. aeruginosa. One example is the loss of the carbapenem porin OprD in P. aeruginosa, which confers carbapenem resistance (8). Similarly, overexpression of the multidrug resistance pump MexAB-OprM increases resistance to meropenem and doripenem. Although imipenem is not a substrate for OprM, elevated AmpC expression increases imipenem resistance. Diverse changes in pump/porin regulation are difficult to comprehensively detect by PCR and were not included in this study.

The NPV and PPV values for prediction of phenotypic resistance from the presence of resistance genes using the generalized linear models depended on the prevalence of resistance in the isolates under evaluation. The NPV and PPV values in Table 4 are for a set of highly resistant bacterial isolates. The NPV and PPV values will increase and decrease, respectively, with a lower prevalence of resistance. For example, Bayes’ theorem estimates an average NPV value of 98% at a 10% prevalence of phenotypic antibiotic resistance across all the combinations of organisms and antibiotics in Table 4. The average corresponding PPV value is 46%. The predictive genotypic models are better at ruling out than ruling in phenotypic antibiotic resistance at a 10% prevalence of resistance and vice versa at a high prevalence of resistance.

The predictive genotypic models are based on phenotypic AST results using the breakpoints for resistant, intermediate, or susceptible phenotypes defined by the MICs set by the Clinical and Laboratory Standards Institute (5). Predictive genotypic models can be generated using the different breakpoints defined by the United States Food and Drug Administration or the European Committee on Antimicrobial Susceptibility Testing. Different breakpoints were not evaluated in this study and are expected to generate different genotypic models with incremental differences in performance.

We used a high-throughput PCR assay and statistical modeling to accurately predict phenotypic antibiotic resistance to several antibiotics based on the presence of resistance genes in clinical isolates of E. coli, K. pneumoniae, P. mirabilis, and P. aeruginosa. Across the various antibiotics, the average sensitivity and specificity for genotypic prediction of phenotypic resistance based on the generalized linear models were 91 and 90%, respectively, for E. coli; 90 and 83%, respectively, for K. pneumoniae; 81 and 89%, respectively, for P. mirabilis; and 85 and 82%, respectively, for P. aeruginosa (Table 4). Note that the specificity value for K. pneumoniae omits the results for cefazolin because all 1,974 isolates were predicted to be resistant to cefazolin. We are testing more carbapenem-resistant E. coli isolates to improve the predictive models for this antibiotic class. Similarly, we are improving the predictive models for P. mirabilis by testing and incorporating another 1,000 isolates of P. mirabilis.

NPV and PPV results for prediction of phenotypic resistance according to the presence of resistance genes depend on the prevalence of resistance and are reported here for a cohort of isolates with a prevalence of resistance higher than that found in typical clinical settings. However, the PPV results from the current study highlight the relative performance of the predictive models across various antibiotics and organisms, while the NPV results highlight the gene menu coverage of various resistance mechanisms across the antibiotics and organisms.

The current retrospective study is large with respect to the number of resistance genes, antibiotics, and bacterial isolates. It also evaluated isolates from multiple types of clinical specimens collected across the world. Our focus on known resistance genes from the scientific literature was one limitation of the study. We are currently evaluating the genotypic predictions of resistance in prospective cohorts of isolates.

PCR is superior to genome sequencing in terms of cost, time to results, and detection of sensitivity with clinical specimens. On the other hand, genome sequencing offers a broader coverage and a higher resolution of antibiotic resistance genes. Several genome sequencing studies of antibiotic resistance have been reported for Staphylococcus aureus and Mycobacterium tuberculosis (9–11), with fewer and more limited studies of Gram-negative organisms being performed. Stoesser et al. (12) reported excellent sensitivity (96%) and specificity (97%) for prediction of phenotypic resistance to amoxicillin, amoxicillin-clavulanic acid, ceftriaxone, ceftazidime, ciprofloxacin, gentamicin, and meropenem based on genome sequencing of 74 E. coli and 69 K. pneumoniae isolates. Similarly, Kos et al. (13) reported on the excellent prediction of phenotypic resistance to meropenem, levofloxacin, and amikacin in 390 clinical isolates of P. aeruginosa through genome sequence analysis of plasmid, chromosomal, and expression-based mechanisms of resistance.

The comprehensive and sensitive detection of antibiotic resistance genes offers the potential to improve patient management by accurately predicting phenotypic resistance for antibiotics often used for front-line empirical therapy of infections caused by Gram-negative bacteria at least a day ahead of growth-based organism identification and phenotypic AST. Our current proof-of-concept study used bacterial isolates to demonstrate the accurate genotypic prediction of phenotypic resistance using a high-throughput multiplex PCR test, which allowed us to genotype more than 7,500 isolates within a reasonable amount of time and at a reasonable cost. Adopting this strategy to detect organisms and predict resistance directly from primary specimens as a culture-independent test has the potential to shorten the time to treatment with appropriate antibiotics and improve patient care. Analysis of several resistance genes also provides bacterial resistance typing for surveillance, infection control, outbreak analysis, and epidemiology studies of multidrug-resistant organisms.

As an extension of the current study, we have commercialized the Acuitas AMR gene panel (for research use only). The test kit is a real-time multiplex PCR test for the semiquantitative detection of E. coli, K. pneumoniae, P. aeruginosa, P. mirabilis, and Enterococcus faecalis plus 47 of the antibiotic resistance genes from Table 3.

## MATERIALS AND METHODS

### Bacterial isolates.

A central microbiology laboratory at the Intermountain Medical Center (Murray, UT) collected and cryopreserved 1,366 clinical isolates of E. coli with an extended-spectrum beta-lactamase (ESBL)-positive phenotype between the years 2008 and 2015. The Intermountain Medical Center central laboratory cultured clinical specimens from 16 acute care hospitals and emergency departments, urgent care clinics, outpatient clinics, two rehabilitation centers, and one long-term acute care hospital during the study period. Clinical and administrative data were not collected, and a random, deidentified convenience sample was submitted for this study. Additional antibiotic-resistant clinical isolates of E. coli, K. pneumoniae, P. mirabilis, and P. aeruginosa were from the SMART study, as supplied by IHMA, Inc., Schaumburg, IL. All bacterial isolates were selected for this study based on previously reported resistance phenotypes without the prior evaluation of resistance genotypes.

### Phenotypic AST.

Phenotypic AST was performed at OpGen on the isolates using a Beckman Coulter MicroScan WalkAway Plus system (Pasadena, CA) and the Neg MIC 45 panel (product number B1017-424), which covers 25 antibiotics. Cryopreserved isolates were subcultured twice on blood agar plates prior to AST. AST results were evaluated using breakpoints for resistant, intermediate, or susceptible phenotypes, as defined by the MICs set by the Clinical and Laboratory Standards Institute (5).

### PCR testing.

The 0.5 McFarland standards were prepared using pure colonies from the same blood agar plates used for AST. Total nucleic acids were extracted from 500 μl of each McFarland standard using a Roche MagNA Pure 96 DNA and Viral NA large-volume kit (product number 06374891001) on a MagNA Pure 96 system (Basel, Switzerland). PCR was performed using primers and fluorescent reporter probes (Applied Biosystems Custom TaqMan MGB probes with 5′-FAM or 5′-VIC with a 3′ nonfluorescent quencher). All PCRs used dUTP instead of TTP along with uracil-DNA glycosylase prior to guard against accidental amplicon contamination. An internal amplification control (a gBlocks gene fragment from Integrated DNA Technologies) was prepared in 1 μg/ml of calf thymus DNA in Tris-EDTA, pH 8 (catalog number BP2473-1; Fisher), and added to all samples to monitor potential PCR inhibition. We also used positive PCR control samples containing gBlocks fragments approximately 1,500 bp in length consisting of concatenated amplicon sequences.

PCR was performed with a Fluidigm BioMark HD system (San Francisco, CA) using 96.96 Dynamic Array IFC arrays, a microfluidic system capable of analyzing 96 samples with 96 separate two-plex PCR assays. Each PCR mixture contained 3 nl of extracted DNA plus 610 nmol/liter each PCR primer, 340 nmol/liter fluorescent reporter probe, and 0.91× Thermo Fisher TaqPath quantitative PCR master mix, CG (product number A16245). Most assays were two-plex PCRs containing two primers and a 6-carboxyfluorescein probe for one target plus two primers and a VIC probe for the other target. PCR was performed with the following cycling program: 2 min at 50°C, 10 min at 95°C, and 40 cycles of 15 s at 95°C and 1 min at 60°C.

Most PCR assays were designed to report the presence of resistance genes, with positive results assigned to PCR assays called positive by the Fluidigm BioMark software with a threshold cycle (*C_T_*) value of between 6 and 40. Some PCR assays were designed to report specific resistance genotypes at the amino acid positions indicated in Table 3, based on relative TaqMan fluorescent intensities from sequence-specific PCR probes (e.g., SHV, TEM, *gyrA*, *parC*).

### Statistical modeling.

Data from the Fluidigm BioMark HD system and MicroScan export files were imported into R (version 3.3.1) software for analysis. BioMark PCR data were tabulated with PCR assays as the columns and isolate identifiers as the rows. PCR *C_T_* values were converted to binary variables, with 1 indicating a positive assay result for that sample. The MicroScan phenotypic data were also converted to binary variables by combining the intermediate and resistant interpretations into a single nonsusceptible state for all the antibiotics, except as noted for cefepime and cefotaxime in Results.

The step function in the statistical packages was used to select predictive PCR assays. This was performed on in-class assays (e.g., assays that detect aminoglycoside-modifying enzyme genes were assessed against the aminoglycoside antibiotic susceptibility phenotype). The R package caret (version 6.0) was used to assess model fits. The train function was used to evaluate 30 iterations of a 10-fold cross validation for each model. When no population-wide predictive combination of PCR assays was found, the relevant assays were examined for individual assays with a strong correlation to phenotypic resistance. These assays were then used in the binary models and act as direct surrogate markers of antibiotic resistance.

## References

[1] Centers for Disease Control and Prevention. 2013 Antibiotic resistance threats in the United States, 2013. Centers for Disease Control and Prevention, Atlanta, GA https://www.cdc.gov/drugresistance/threat-report-2013/pdf/ar-threats-2013-508.pdf.

[2] Centers for Disease Control and Prevention. 2015 Facility guidance for control of carbapenem-resistant *Enterobacteriaceae* (CRE): November 2015 update - CRE toolkit. Centers for Disease Control and Prevention, Atlanta, GA https://www.cdc.gov/hai/pdfs/cre/cre-guidance-508.pdf.

[3] Pancholi P, Carroll KC, Buchan BW, Chan RC, Dhiman N, Ford B, Granato PA, Harrington AT, Hernandez DR, Humphries RM, Jindra MR, Ledeboer NA, Miller SA, Mochon AB, Morgan MA, Patel R, Schreckenberger PC, Stamper PD, Simner PJ, Tucci NE, Zimmerman C, Wolk DM 2018 Multicenter evaluation of the Accelerate PhenoTest BC kit for rapid identification and 2 phenotypic antimicrobial susceptibility testing using morphokinetic cellular analysis. J Clin Microbiol 56:e01329-17. doi:10.1128/JCM.01329-17. PMC5869823 29305546

[4] Baltekin O, Boucharin A, Tano E, Andersson DI, Elf J 2017 Antibiotic susceptibility testing in less than 30 min using direct single-cell imaging. Proc Natl Acad Sci U S A 114:9170–9175. doi:10.1073/pnas.1708558114. 28790187 PMC5576829

[5] CLSI. 2017 Performance standards for antimicrobial susceptibility testing, 27th ed. CLSI supplement M100. Clinical and Laboratory Standards Institute, Wayne, PA.

[6] Liu B, Pop M 2009 ARDB—Antibiotic Resistance Genes Database. Nucleic Acids Res 37:D443–D447. doi:10.1093/nar/gkn656. 18832362 PMC2686595

[7] McArthur AG, Waglechner N, Nizam F, Yan A, Azad MA, Baylay AJ, Bhullar K, Canova MJ, De Pascale G, Ejim L, Kalan L, King AM, Koteva K, Morar M, Mulvey MR, O'Brien JS, Pawlowski AC, Piddock LJ, Spanogiannopoulos P, Sutherland AD, Tang I, Taylor PL, Thaker M, Wang W, Yan M, Yu T, Wright GD 2013 The Comprehensive Antibiotic Resistance Database. Antimicrob Agents Chemother 57:3348–3357. doi:10.1128/AAC.00419-13. 23650175 PMC3697360

[8] Davies TA, Marie Queenan A, Morrow BJ, Shang W, Amsler K, He W, Lynch AS, Pillar C, Flamm RK 2011 Longitudinal survey of carbapenem resistance and resistance mechanisms in Enterobacteriaceae and non-fermenters from the USA in 2007-09. J Antimicrob Chemother 66:2290–2307. doi:10.1093/jac/dkr290. 21775338

[9] Gordon NC, Price JR, Cole K, Everitt R, Morgan M, Finney J, Kearns AM, Pichon B, Young B, Wilson DJ, Llewelyn MJ, Paul J, Peto TEA, Crook DW, Walker AS, Golubchik T 2014 Prediction of *Staphylococcus aureus* antimicrobial resistance by whole-genome sequencing. J Clin Microbiol 52:1182–1191. doi:10.1128/JCM.03117-13. 24501024 PMC3993491

[10] Bradley P, Gordon NC, Walker TM, Dunn L, Heys S, Huang B, Earle S, Pankhurst LJ, Anson L, de Cesare M, Piazza P, Votintseva AA, Golubchik T, Wilson DJ, Wyllie DH, Diel R, Niemann S, Feuerriegel S, Kohl TA, Ismail N, Omar SV, Smith EG, Buck D, McVean G, Walker AS, Peto TEA, Crook DW, Iqbal A 2015 Rapid antibiotic-resistance predictions from genome sequence data for *Staphylococcus aureus* and *Mycobacterium tuberculosis*. Nat Commun 6:10063. doi:10.1038/ncomms10063. 26686880 PMC4703848

[11] Schleusener V, Koser CU, Beckert P, Niemann S, Feuerriegel S 2017 Mycobacterium tuberculosis resistance prediction and lineage classification from genome sequencing: comparison of automated analysis tools. Sci Rep 7:46327. doi:10.1038/srep46327. 28425484 PMC7365310

[12] Stoesser N, Batty EM, Eyre DW, Morgan M, Wyllie DH, Del Ojo Elias C, Johnson JR, Walker AS, Peto TEA, Crook DW 2013 Predicting antimicrobial susceptibilities for *Escherichia coli* and *Klebsiella pneumoniae* isolates using whole genomic sequence data. J Antimicrob Chemother 68:2234–2244. doi:10.1093/jac/dkt180. 23722448 PMC3772739

[13] Kos VN, Déraspe M, McLaughlin RE, Whiteaker JD, Roy PH, Alm RA, Corbeil J, Gardner H 2015 The resistome of *Pseudomonas aeruginosa* in relationship to phenotypic susceptibility. Antimicrob Agents Chemother 59:427–436. doi:10.1128/AAC.03954-14. 25367914 PMC4291382

